# Oligomerization of the Vesicular Stomatitis Virus Phosphoprotein Is Dispensable for mRNA Synthesis but Facilitates RNA Replication

**DOI:** 10.1128/JVI.00115-20

**Published:** 2020-06-16

**Authors:** Louis-Marie Bloyet, Benjamin Morin, Vesna Brusic, Erica Gardner, Robin A. Ross, Tegy Vadakkan, Tomas Kirchhausen, Sean P. J. Whelan

**Affiliations:** a Department of Microbiology, Harvard Medical School, Boston, Massachusetts, USA; b Program in Cellular and Molecular Medicine, Boston Children’s Hospital and Department of Cell Biology, Harvard Medical School, Boston, Massachusetts, USA; c Department of Molecular Microbiology, Washington University in St. Louis, St. Louis, Missouri, USA; University of Texas Southwestern Medical Center

**Keywords:** genome replication, oligomerization, phosphoprotein, polymerase, vesicular stomatitis virus

## Abstract

Nonsegmented negative-strand (NNS) RNA viruses possess a ribonucleoprotein template in which the genomic RNA is sequestered within a homopolymer of nucleocapsid protein (N). The viral RNA-dependent RNA polymerase (RdRP) resides within an approximately 250-kDa large protein (L), along with unconventional mRNA capping enzymes: a GDP:polyribonucleotidyltransferase (PRNT) and a dual-specificity mRNA cap methylase (MT). To gain access to the N-RNA template and orchestrate the L_RdRP_, L_PRNT_, and L_MT_, an oligomeric phosphoprotein (P) is required. Vesicular stomatitis virus (VSV) P is dimeric with an oligomerization domain (OD) separating two largely disordered regions followed by a globular C-terminal domain that binds the template. P is also responsible for bringing new N protomers onto the nascent RNA during genome replication. We show VSV P lacking the OD (P_ΔOD_) is monomeric but is indistinguishable from wild-type P in supporting mRNA transcription *in vitro*. Recombinant virus VSV-P_ΔOD_ exhibits a pronounced kinetic delay in progeny virus production. Fluorescence recovery after photobleaching demonstrates that P_ΔOD_ diffuses 6-fold more rapidly than the wild type within viral replication compartments. A well-characterized defective interfering particle of VSV (DI-T) that is only competent for RNA replication requires significantly higher levels of N to drive RNA replication in the presence of P_ΔOD_. We conclude P oligomerization is not required for mRNA synthesis but enhances genome replication by facilitating RNA encapsidation.

**IMPORTANCE** All NNS RNA viruses, including the human pathogens rabies, measles, respiratory syncytial virus, Nipah, and Ebola, possess an essential L-protein cofactor, required to access the N-RNA template and coordinate the various enzymatic activities of L. The polymerase cofactors share a similar modular organization of a soluble N-binding domain and a template-binding domain separated by a central oligomerization domain. Using a prototype of NNS RNA virus gene expression, vesicular stomatitis virus (VSV), we determined the importance of P oligomerization. We find that oligomerization of VSV P is not required for any step of viral mRNA synthesis but is required for efficient RNA replication. We present evidence that this likely occurs through the stage of loading soluble N onto the nascent RNA strand as it exits the polymerase during RNA replication. Interfering with the oligomerization of P may represent a general strategy to interfere with NNS RNA virus replication.

## Abstract (precis)

All NNS RNA viruses, including the human pathogens rabies, measles, respiratory syncytial virus, Nipah, and Ebola, possess an essential L-protein cofactor, required to access the N-RNA template and coordinate the various enzymatic activities of L. The polymerase cofactors share a similar modular organization of a soluble N-binding domain and a template-binding domain separated by a central oligomerization domain. Using a prototype of NNS RNA virus gene expression, vesicular stomatitis virus (VSV), we determined the importance of P oligomerization. We find that oligomerization of VSV P is not required for any step of viral mRNA synthesis but is required for efficient RNA replication. We present evidence that this likely occurs through the stage of loading soluble N onto the nascent RNA strand as it exits the polymerase during RNA replication. Interfering with the oligomerization of P may represent a general strategy to interfere with NNS RNA virus replication.

## INTRODUCTION

The 241-kDa large (L) protein of vesicular stomatitis virus (VSV) contains all the enzymatic activities necessary to copy its viral ribonucleoprotein (RNP) template (1–4). That RNP template comprises the genomic RNA completely encased within the viral nucleocapsid (N) protein (5, 6). Responding to specific RNA signals, the RNA-dependent RNA polymerase of L (L_RdRP_) transcribes the RNP template into a 47-nucleotide (nt) 5′ triphosphate leader RNA and 5 monocistronic mRNAs that are indistinct to host cell mRNA with respect to their 5′ cap and 3′ polyadenylate (7–10). The enzymatic activities necessary for formation of the viral 5′ mRNA cap structure reside within L and differ markedly from those of the host cell and other organisms (11). During viral mRNA cap formation, a GDP:polyribonucleotidyltransferase (L_PRNT_) transfers the 5′ end of the nascent RNA chain onto a GDP acceptor through a covalent L-pRNA (L-monophosphate RNA) intermediate. The resulting GpppA cap structure is then modified by a dual-specificity L-encoded RNA cap methylase (L_MT_), which first modifies the ribose-2′-O position associated with first transcribed nucleotide generating GpppAm and subsequently methylate, the guanine ring of the cap at the N7 position, to yield 7mGpppAm (12). The 3′ polyadenylate tail is generated by a chattering mechanism in which the L_RdRP_ reiteratively copies one or more members of an oligo(U) tract resident at the end of each gene (13).

The RNP template is also used to generate full-length genomic RNPs through an antigenomic RNP intermediate. RNA replication requires the L_RdRP_ and a continuous supply of soluble N protein that coats the nascent RNA chain during its synthesis (14). Although the mRNA capping enzymes are not required during replication, as the 5′ end of the replication products are unmodified triphosphate, this process of terminal initiation depends on a priming loop donated from the L_PRNT_ into the L_RdRP_ (4, 15). Despite containing all of the enzymes necessary for RNA synthesis, L cannot copy the N-RNA template without forming a complex with its cofactor, a 29-kDa oligomeric phosphoprotein (P) (1). Complex formation of L with P plays a key role in also organizing the various enzymatic domains of L with respect to one another (16). This defines the minimal viral machinery for RNA synthesis as the N-RNA template and the viral P and L proteins.

Structural studies of the VSV replication machinery highlight the master role of P in orchestrating RNA synthesis. The genomic RNA is completely coated by N, which must be transiently dissociated during copying by L. A globular C-terminal domain of P (P_CTD_), corresponding to residues 195 to 265, contacts the N-RNA template by binding at a unique interface that forms between adjacent N molecules on the template (17). That P_CTD_ is separated from a largely disordered N-terminal region of P by an oligomerization domain (P_OD_) comprising residues 107 to 177 (18). Although the N-terminal domain of P is largely disordered, complex formation with L defines key contacts between L and two segments of P encompassing residues 49 to 56 and 82 to 105 (19). A short stretch of the N terminus of P (residues 5 to 34) also binds a groove between the N- and C-terminal lobes of monomeric N (N^0^), occluding binding of RNA (20). This likely reflects how soluble N protein is loaded onto the nascent RNA chain during RNP assembly that is concomitant with viral genome replication.

The central role of P in regulating gene expression prompted us to probe the requirement for its oligomerization in VSV. We deleted the oligomerization domain of P, validated that the resulting protein is monomeric, interrogated the consequences for gene expression *in vitro* and in cells, and generated a recombinant virus lacking P_OD_. The data demonstrate that viral mRNA synthesis is unaffected by P protein oligomerization, and they identify a pronounced kinetic delay in viral RNA replication for monomeric P. Probing of the underlying mechanism behind this delay demonstrates that the oligomerization of P facilitates genome RNA replication at a lower N protein concentration, likely by directly influencing the loading of N onto the nascent transcript. This work defines a key function for oligomerization of the P protein of VSV that likely extends to other viruses in the order *Mononegavirales*.

## RESULTS

### VSV P lacking its oligomerization domain is monomeric in solution.

**FIG 1:**
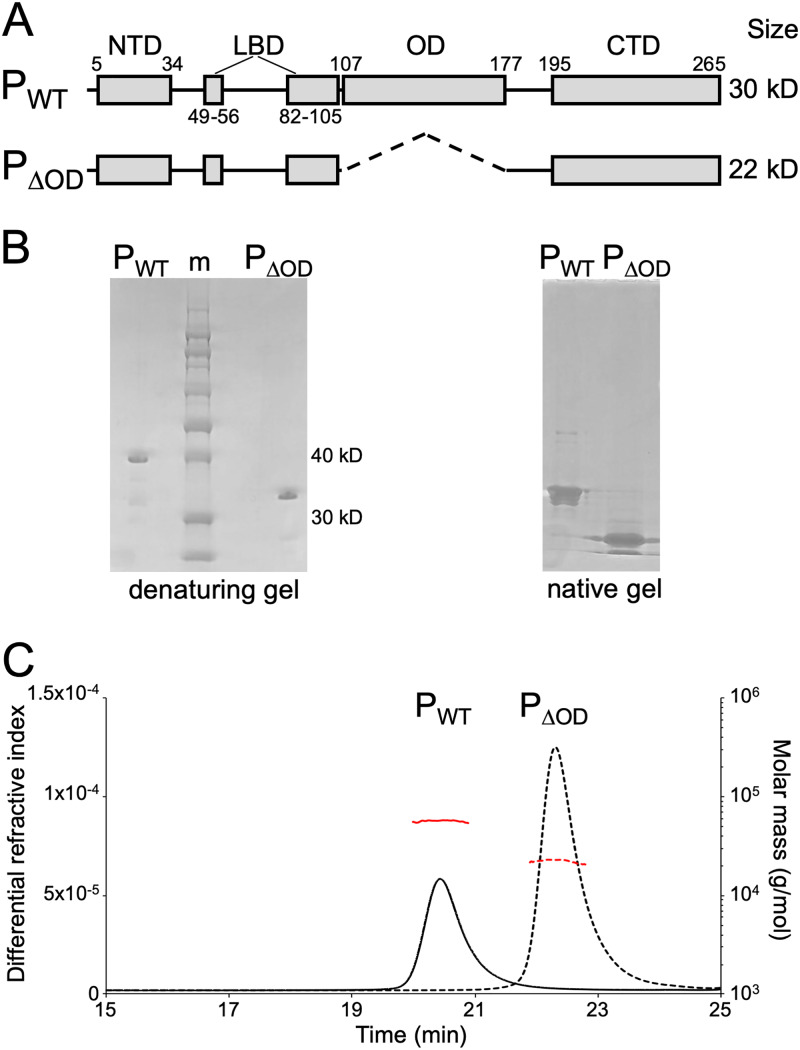
Characterization of P_ΔOD_. (A) Schematic of VSV P_WT_ and P_ΔOD_ showing their modular organization with the N-terminal domain (NTD), L-binding domains (LBD), oligomerization domain (OD), and C-terminal domain (CTD) represented as rectangles. (B) Analysis of purified P_WT_ and P_ΔOD_ proteins by polyacrylamide gel electrophoresis on a denaturing gel (left) and a native gel (right). Sizes of two bands of the protein ladder (m) are indicated. (C) SEC-MALS analysis of purified P_WT_ (solid lines) and P_ΔOD_ (dashed lines) proteins. The horizontal red traces show the inferred molecular mass. Predicted molecular masses for monomeric and dimeric P_WT_ are 30.3 kDa and 60.6 kDa, respectively. For monomeric and dimeric P_ΔOD_, predicted molecular masses are 22.4 kDa and 44.8 kDa, respectively. Observed experimental molecular masses are 56.2 kDa and 22.6 kDa for P_WT_ and P_ΔOD_, respectively.

To probe the function of P protein oligomerization in assembly and function of the VSV replication machinery, we expressed and purified from bacteria a variant of P lacking residues 107 to 177, termed P_ΔOD_ (Fig. 1A and B). Comparison of identically prepared wild-type P (P_WT_) and P_ΔOD_ on denaturing and native protein gels reveals that deletion of the oligomerization domain yields a P variant whose mobility is altered, consistent with the loss of the 8-kDa OD (Fig. 1B). To provide evidence that P_ΔOD_ is present as a monomer in solution, we used size exclusion chromatography and multiple-angle laser light scattering (SEC-MALS). Both P_WT_ and P_ΔOD_ elute as single monodisperse peaks (Fig. 1C) and allowed us to calculate the molar mass of the corresponding protein. For P_WT_, the measured weight-averaged molar mass of 56.2 kDa was within the range of the expected mass of a full-length P dimer (60.6 kDa). The weight-average molar mass (22.6 kDa) for P_ΔOD_ is in good agreement with the expected molar mass for a monomer (22.4 kDa). Thus, deletion of the oligomerization domain of VSV P renders the protein monomeric in solution.

### P_ΔOD_ is functional for RNA synthesis *in vitro*.

**FIG 2:**
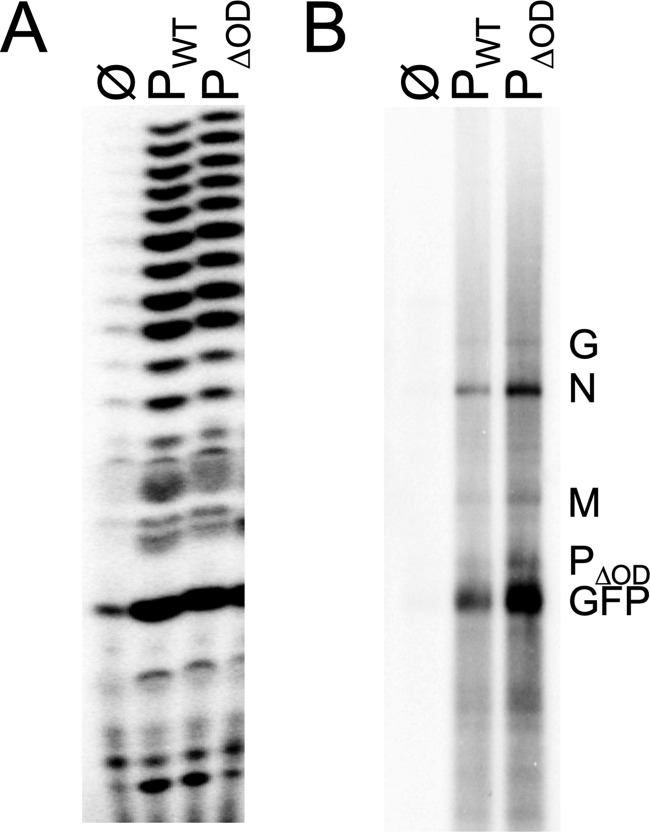
Functional analysis of P_ΔOD_ *in vitro*. Reactions were performed in the absence (Ø) or presence of equimolar amounts of P_WT_ or P_ΔOD_ using purified L and either a synthetic, naked 19-nt RNA template corresponding to the 3′ leader sequence of the VSV genome (A) or a purified, encapsidated N-RNA template (B). Radioactive products were analyzed by gel electrophoresis on a 20% acrylamide gel (A) or a 1.75% acid-agarose gel containing 6 M urea (B). (A) *n* = 1 replicate; (B) representative experiment (*n* = 3 replicates).

We next compared the ability of P_WT_ and P_ΔOD_ to stimulate L_RdRP_ activity using an *in vitro* assay with chemically synthesized RNA as the template (21). At equimolar amounts, P_ΔOD_ and P_WT_ stimulate the L_RdRP_ indistinguishably (Fig. 2A). The natural template for RNA synthesis is, however, the genomic RNA completely encased in a nucleocapsid protein sheath. To compare the ability of P_WT_ and P_ΔOD_ to facilitate L-mediated copying of the natural template, we isolated N-RNA from virions and reconstituted RNA synthesis *in vitro*. Briefly, N-RNA is incubated with purified L and equimolar amounts of P_WT_ or P_ΔOD_, and RNA synthesis assessed by incorporation of [^32^P]GTP with subsequent analysis of the products on acid-agarose gels (Fig. 2B). The results of this analysis demonstrate that both P_ΔOD_ and P_WT_ have the ability to produce the 5 viral mRNAs, confirming that the oligomerization domain of P is not required for mRNA synthesis. We observed, however, an apparent increase in mRNAs produced by P_ΔOD_, and this was seen consistently among experiments.

### A recombinant VSV lacking the oligomerization domain of P is defective in viral multiplication.

**FIG 3:**
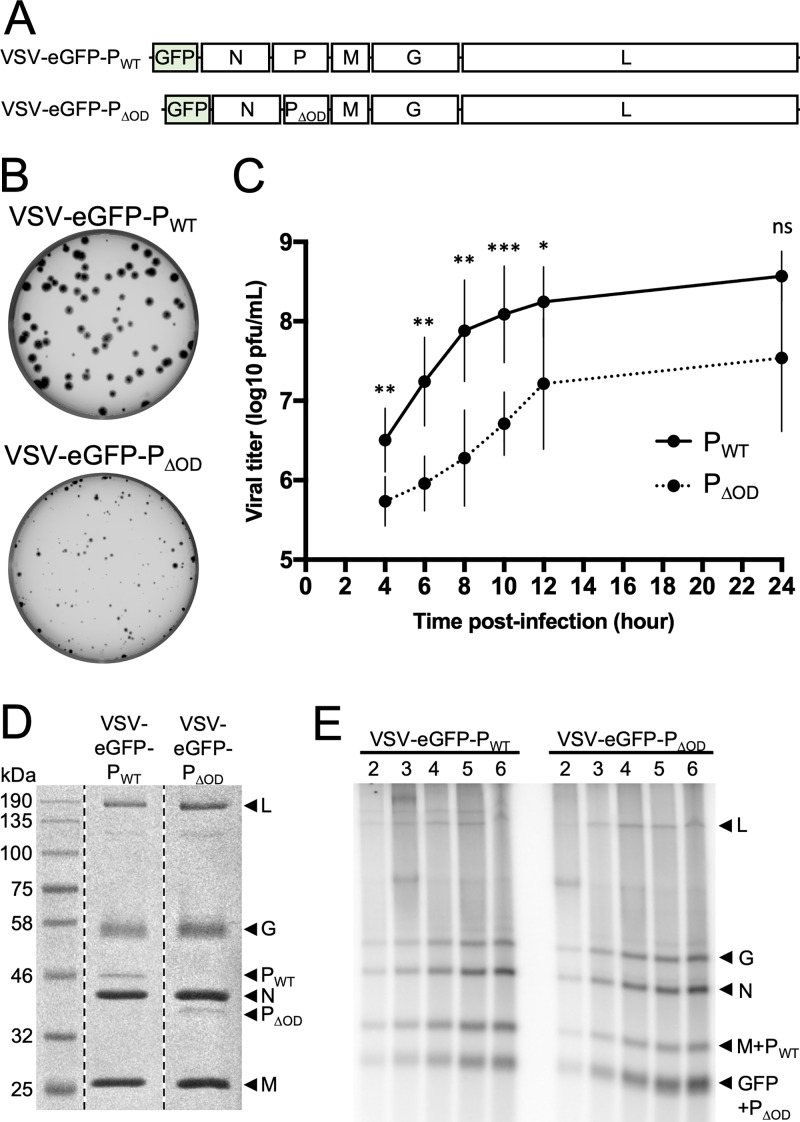
Characterization of a recombinant VSV expressing P_ΔOD_. (A) Schematic representation of the recombinant virus genomes. (B) Viral spreading as seen by plaque assay on Vero cells infected with VSV-eGFP-P_WT_ or VSV-eGFP-P_ΔOD_. (C) Viral growth kinetic on Vero cells infected with VSV-eGFP-P_WT_ (P_WT_, black line) or VSV-eGFP-P_ΔOD_ (P_ΔOD_, dotted line) at an MOI of 3. Supernatants were harvested and titers determined at 4, 6, 8, 10, 12, and 24 h postinfection. Statistical analysis was performed by a paired *t* test. * *P*, < 0.05; ** *P*, < 0.005; *** *P*, < 0.0005; ns, nonsignificant. (D) Analysis of virion protein content. Gradient-purified virions (10^8^ PFU) were denaturated by SDS and heat and analyzed by SDS-PAGE and Coomassie staining. (E) In phosphate-free media supplemented with radioactive [^32^P]orthophosphate, BSR-T7 cells were treated with 10 μg/ml actinomycin D and 100 μg/ml cycloheximide and infected at an MOI of 100 with VSV-eGFP-P_WT_ or VSV-eGFP-P_ΔOD_. RNA was extracted at 2, 3, 4, 5, and 6 h postinfection and analyzed on a 1.75% acid-agarose gel containing 6 M urea. Representative experiment (*n* = 4).

To examine whether oligomerization of P is required for viral amplification in cell culture, we deleted residues 107 to 177 of P in an infectious molecular clone of the virus that expresses enhanced green fluorescent protein (eGFP) as a marker of infection (Fig. 3A). The resulting virus, VSV-eGFP-P_ΔOD_, exhibits a growth defect in cell culture, as is evident by both its plaque size (Fig. 3B) and a 4-h delay before the onset of the exponential phase of virion production in a single-step growth assay (Fig. 3C). Molecular characterization of the virus revealed that the genomic sequence retained the desired deletion and that the composition of purified VSV-eGFP-P_ΔOD_ virions is indistinct to those of VSV-eGFP-P_WT_ by SDS-PAGE with respect to the relative amounts of the 5 viral proteins and the particle-to-PFU ratio (Fig. 3D). This result indicates that ablation of the oligomerization domain of P has little effect on protein incorporation into particles but attenuates viral growth in cell culture.

To begin to understand the mechanism underlying the kinetic delay in viral multiplication, we compared the ability of VSV-eGFP-P_WT_ and VSV-eGFP-P_ΔOD_ to undertake primary transcription in cells. Cells were treated with cycloheximide and actinomycin D to block protein and cellular RNA synthesis, respectively, and infected with the indicated virus at a multiplicity of infection (MOI) of 100, and viral RNA synthesis was monitored by metabolic incorporation of [^32^P]orthophosphate. At various times postinfection, total cellular RNA was extracted and analyzed by acid-agarose gel electrophoresis, and the products of primary transcription were detected by phosphor image analysis. The abundance of each viral mRNA was similar for VSV-eGFP-P_WT_ and VSV-eGFP-P_ΔOD_ (Fig. 3E), demonstrating that loss of the oligomerization domain of P does not compromise transcription. Consistent with the experiments of Fig. 2B, VSV-eGFP-P_ΔOD_ primary transcription appears to generate more mRNA than VSV-eGFP-P_WT_. Quantitation of the N mRNA signal coupled with statistical analysis yields a *P* value of 0.0562 (Fig. 3E). This result demonstrates that the polymerase packaged into particles remains associated with the N-RNA template in the absence of the P_OD_ and that there is no apparent consequence for the stability of the polymerase complex. Furthermore, because the relative proportions of the viral mRNAs are indistinct, P_OD_ is not required to retain the L protein on the template as it navigates gene junctions. These data imply that a step downstream of transcription, likely RNA replication, is responsible for the observed kinetic delay in viral growth.

### The oligomerization domain of P affects protein diffusion in viral replication compartments.

**FIG 4:**
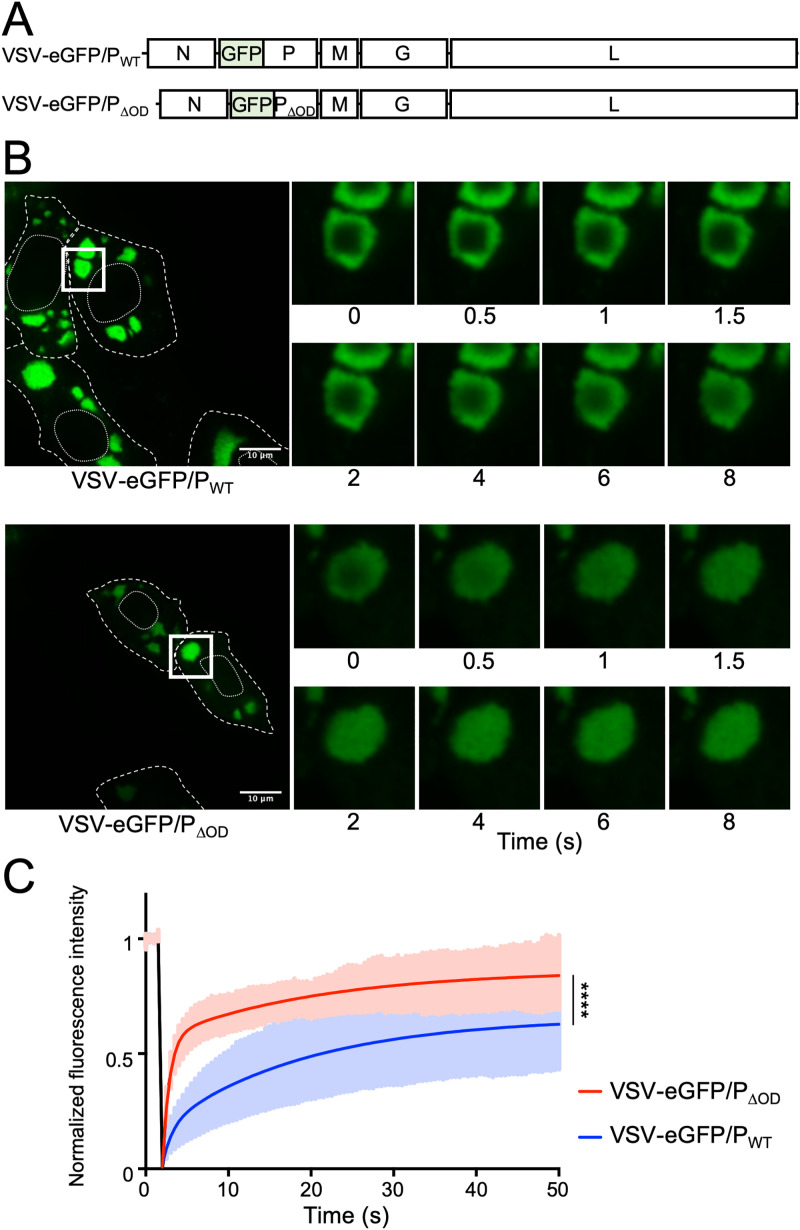
FRAP analysis of replication compartments. (A) Schematic representation of the recombinant virus genomes. (B, C) Vero cells were infected at an MOI of 3 with VSV-eGFP/P_WT_ or VSV-eGFP/P_ΔOD_, and eGFP was visualized with a spinning disk confocal microscope at 6 and 10 h postinfection for VSV-eGFP/P_WT_ and VSV-eGFP/P_ΔOD_, respectively. Fluorescence recovery after photobleaching (FRAP) experiments were performed on areas of 4 μm^2^ located inside compartments. Recovery fluorescence was measured every 500 ms for 50 s. (B) Infected cells before photobleaching (left), and zoomed-in pictures taken at indicated times after photobleaching (right). Dashed and dotted lines delimit the cells and the nucleus, respectively. Squares represent the zoomed-in sections. (C) FRAP data were corrected for background, normalized to the minimum and maximum intensities. The mean is shown on the black line, with the gray zone representing the SD. Mean experimental curves were fitted with double-exponential models (red line; VSV-eGFP/P_WT_, *R^2^* = 0.997; VSV-eGFP/P_ΔOD_, *R^2^* = 0.996). Statistical comparison of the two data sets was performed using the Kolmogorov-Smirnov test. *P* < 0.0001.

Following infection of cells, the viral replication machinery is found in the cytoplasm in a compartment that is not bounded by a membrane (22, 23). Work with rabies virus implicates the oligomerization domain of its phosphoprotein as required for the reconstitution of such compartments (24). We therefore generated a recombinant virus expressing P_ΔOD_ fused to eGFP at its N terminus (termed VSV-eGFP/P_ΔOD_), which allows visualization of the replication compartment (Fig. 4A). The eGFP/P viruses differ from eGFP-P, which encodes an eGFP reporter from a separate transcription unit inserted between the leader and N genes. As seen for wild-type P, we found the eGFP/P_ΔOD_ concentrates in structures that look morphologically similar (Fig. 4B). We next compared the properties of the compartments by measuring fluorescence recovery after photobleaching (FRAP) of eGFP/P. To take into account the growth delay of VSV-eGFP-P_ΔOD_, experiments were performed at 6 and 10 hpi for VSV-eGFP/P_WT_ and VSV-eGFP/P_ΔOD_, respectively. Previous work demonstrated that the size of viral replication compartments impacts the diffusion properties of the viral proteins (25). We therefore selected similar-sized structures for this analysis which were present in cells infected with VSV-eGFP/P_WT_ at 6 hpi and cells infected with VSV-eGFP/P_ΔOD_ at 10 hpi. We measured a half-maximal recovery time of 12.3 s for eGFP/P_WT_ containing compartments compared with 2.1 s for compartments containing eGFP/P_ΔOD_ (Fig. 4B and C). This result demonstrates that P_ΔOD_ recovers more rapidly from photobleaching, which means that the protein must undergo a more rapid diffusion and exchange of P_ΔOD_ than wild-type P. This is consistent with the expected reduction in intermolecular interactions following ablation of the oligomerization domain.

### RNA replication requires higher N protein concentrations for monomeric P.

**FIG 5:**
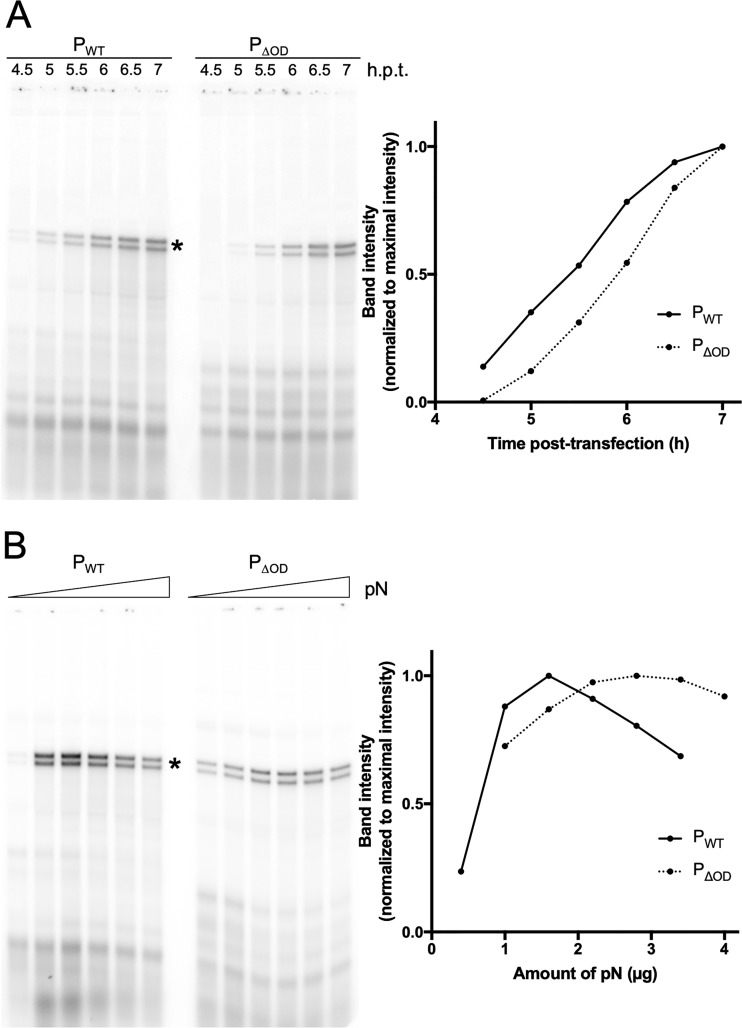
Effect of P_ΔOD_ on viral RNA synthesis. (A) BSR-T7 cells were infected with DI-T for 1 h and transfected with plasmids coding for L, N, and P_WT_ or P_ΔOD_. At 1.5, 2, 2.5, 3, 3.5, or 4 h posttransfection, cells were incubated for 3 h in phosphate-free media supplemented with radioactive [^32^P]orthophosphate and 10 μg/ml actinomycin D. RNA was harvested and analyzed on a 1.75% agarose gel containing 6 M urea (left). DI-T band intensities were quantified and plotted as percentage of maximal intensity (right). Times of harvest posttransfection are indicated. DI-T bands are marked with an asterisk. Representative experiment (*n* = 4). (B) BSR-T7 cells were infected with DI-T for 1 h and transfected with plasmids coding for L, N, and P_WT_ or P_ΔOD_. Increasing amounts of plasmid coding for N were transfected with 0.4, 1, 1.6, 2.2, 2.8, and 3.4 μg for P_WT_ and 1, 1.6, 2.2, 2.8, 3.4, and 4 μg for P_ΔOD_. Five hours posttransfection, cells were incubated for 3 h in phosphate-free media supplemented with radioactive [^32^P]orthophosphate and 10 μg/ml actinomycin D. RNA was harvested and analyzed on a 1.75% agarose gel containing 6 M urea (left). DI-T band intensities were quantified and plotted as percentage of maximal intensity (right). Representative experiment (*n* = 2).

Viral genome replication is obligatorily coupled to encapsidation of the nascent RNA chain by soluble nucleocapsid proteins, and available evidence underscores the central role of P in that assembly process (26, 27). To directly measure the influence of the oligomerization domain of P on RNA replication, we employed a cell-based assay of RNA replication for a well-characterized defective interfering (DI) particle of VSV, DI-T (28). The genome of DI-T contains a 5′ copyback arrangement in which the antigenomic promoter, which exclusively supports replication, drives synthesis from both the genomic and antigenomic strands. In this assay, BSR-T7 cells are infected with DI-T particles and transfected with different amounts of plasmids expressing L, N, and P_WT_ or P_ΔOD_. The products of RNA replication are then directly detected by metabolic incorporation of radioactive orthophosphate in the presence of actinomycin D, purified, and analyzed by acid-agarose electrophoresis (Fig. 5A). The acid-agarose gels facilitate the separation of the positive and negative strands of the DI genome RNA, allowing us to measure both genome and antigenome synthesis. Quantitation of the DI-T genome and antigenome bands demonstrates that they increase over time at a similar rate and reach the same levels for P_WT_ and P_ΔOD_, but it highlights a delay in accumulation of replication products for P_ΔOD_ (Fig. 5A).

We next hypothesized that the oligomerization domain of P could serve to concentrate N during the process of nascent strand encapsidation, which would suggest that a higher concentration of N would further stimulate replication for P_ΔOD_. It was previously established that altering the concentration of N plasmid transfected into cells alters the concentration of N protein and that there is an optimal N-P ratio to support maximal replication (29). The optimal N plasmid concentration to support maximal replication is higher for P_ΔOD_ than P_WT_, consistent with higher concentrations of N protein being required for replication (Fig. 5B). Together with the time course experiment, these results suggest that P oligomerization plays a role in genome replication by lowering the N concentration threshold required to initiate the replication phase of the cycle.

## DISCUSSION

The major conclusions of this study are (i) the oligomerization domain of VSV P results in its dimerization, (ii) P dimerization is dispensable for all aspects of viral mRNA transcription, and (iii) P dimerization stimulates RNA replication. We also present evidence in support of a mechanism where the dimerization-mediated stimulation of replication is likely accomplished by facilitating encapsidation of the nascent replicative product at an optimal N protein concentration that is lowered by the presence of the oligomerization domain. We suggest that this is a central function of the P proteins of all *Mononegavirales* and that interfering with the oligomeric state of P may serve as a general mechanism to inhibit replication of these viruses.

### VSV P protein dimerization.

The oligomeric status of VSV P has been variably reported as trimer (30), tetramer (31), and, most recently, following structural studies of the oligomerization domain, as a dimer (18, 32). The SEC-MALS data presented here provide support that P exists as a homodimer and that homodimerization is mediated by the oligomerization domain. During the preparation of the manuscript, Gérard and colleagues published structural analyzes of purified P_ΔOD_ confirming P_ΔOD_ is monomeric in solution and showing its global architecture is not affected by the loss of the OD (33). In addition to forming a homodimer, P has additional viral binding partners, including N-RNA, N^0^, and L. Using electron cryomicroscopy, we recently defined key contacts between L and two regions of the P N-terminal domain (P_NTD_) (19). In that structure, however, P is noncontiguous; thus, we cannot exclude the possibility that 2 separate molecules of P can bind L. Crystallographic studies showed the P_CTD_ binds the N-RNA at an interface only formed by adjacent N protomers (17). The engagement of the N-RNA by the P_CTD_ raises the possibility that even in the absence of an oligomerization domain, P may be able to effectively function as an oligomer with respect to L through its template-binding properties. Additional structural studies are likely to help unravel this question.

### Virion production.

Since the production of infectious viral particles is delayed for a virus expressing P_ΔOD_, one or several of the viral cycle steps, including viral entry, primary transcription, genome replication, or particle assembly, must be affected. Using a similar recombinant virus, Gérard and colleagues did not see any strong effect of the loss of the OD on virion production (33). We cannot explain the different conclusion of this earlier study, but this may reflect strain differences or the use of different assays between our studies. Our conclusion that the loss of the OD affects viral replication by influencing a key step involved in viral RNA replication and genome encapsidation comes from biochemical analysis of the RNA products synthesized from a naturally occurring defective interfering particle of VSV. This assay differs from the use of synthetic minigenome in the earlier work. Additional work will be required to understand the basis for this distinction.

### Viral replication compartments.

For rabies virus, the P_OD_ is reported to be essential for the formation of the viral replication compartment in infected cells (24). This is not the case for VSV-eGFP/P_ΔOD_, which still forms compartments. While such compartments form in cells infected with VSV-eGFP/P_ΔOD_, the properties of the compartments differ with respect to the diffusion of eGFP/P_WT_, as evidenced by a 6-fold faster recovery rate following photobleaching. We interpret that increased recovery rate as reflective of the loss of the oligomerization domain, effectively decreasing the number of partners for P in its monomeric versus dimeric states, and that those interactions contribute to the slower recovery observed in wild-type infected cells. As the properties of inclusions alter with their size, which itself is related to the stage of the infectious cycle, we cannot exclude that the difference in P diffusion might also be linked to the acquisition of the data at 6 versus 10 hours postinfection (hpi) rather than intrinsic differences between P_WT_ and P_ΔOD_.

### Viral transcription.

Reconstitution of transcription *in vitro* on naked RNA or on purified N-RNA templates was unaffected by the loss of the OD. Consistent with this observation, measures of primary mRNA transcription by the polymerase molecules brought into cells with the infecting virus were also unaffected. Collectively, those data provide compelling evidence that no step of transcription, including initiation, cap formation, elongation, polyadenylation, termination, and reinitiation, require the presence of the OD. We consistently observed an increase in levels of transcription with P_ΔOD_, although quantitation of the N mRNA indicated that this difference was not significant. Additional work will be required to understand the basis of this observation. An earlier model of migration of the polymerase complex of Sendai virus, a related virus in the family *Paramyxoviridae*, posited that the polymerase cartwheels along the N-RNA template via the interaction between the P_CTD_ of its tetrameric P and the nucleocapsid (34). Such a cartwheeling model suggests that dissociation of the polymerase at gene junctions would be favored when interactions between P and the N-RNA template are compromised. Studies with measles virus found that modulation of the interaction between its P_CTD_ and the N-RNA alters the relative abundance of viral mRNAs (35, 36). In contrast, deletion of the oligomerization domain of VSV P does not alter the gradient of transcripts produced from the template, suggesting that the interaction between the L protein and template is not compromised. This suggests that either modifying the strength of the VSV P_CTD_-N-RNA interaction by tuning its affinity or its avidity has different outcomes, or the interaction of VSV P_CTD_ with the N-RNA is not a key determinant of transcription reinitiation at gene junctions.

### RNA replication.

During RNA replication, in addition to the requirements for P as a cofactor for the polymerase and its role in binding the N-RNA template, P loads soluble N protomers onto the nascent RNA (26, 27). The availability of a pool of N^0^-P for loading onto that nascent strand is thought to regulate polymerase activity during leader synthesis, coupling the nascent strand encapsidation to genome replication (14, 37). Absent robust assays for the *in vitro* reconstitution of RNA encapsidation, direct tests of the role of the P_OD_ in encapsidation are not possible, and therefore, in this study, we used a cell-based assay that reports on encapsidation indirectly by measuring the accumulation of the products of RNA replication. Using that assay, we attained evidence that when equal amounts of N and L are available, the kinetics with which RNA replication is established with P_ΔOD_ are slower. We also found that replication is further stimulated by increasing the amount of N in cells through increasing levels of an N expression plasmid. The structure of the VSV L-P complex reveals that the region of P that binds N^0^ is positioned by the RNA exit channel from the polymerase poised to encapsidate the nascent RNA strand (19). We cannot exclude the possibility that the OD itself plays a role during the replication, nor that changing the spatial arrangement of the N-terminal and C-terminal domains impairs proper function. However, as both P_NTD_ of a P_WT_ dimer can bind simultaneously an N monomer, perhaps dimerization of P facilitates a local increase in the concentration of N^0^ at the site of encapsidation favoring RNA replication (32). As P_WT_ is a dimer, its interaction with N^0^ is likely enhanced by avidity, thus potentially further increasing the recruitment of N^0^ at the encapsidation site. Robust *in vitro* encapsidation assays and structural studies of polymerase complexes during RNA replication will likely prove informative.

## MATERIALS AND METHODS

### Cells.

BSR-T7 cells (a kind gift from K. Conzelmann) (38) and African green monkey kidney Vero cells (ATCC CCL-81) were maintained in Dulbecco’s modified Eagle’s medium (DMEM; Corning Inc.; product no. 10-013-CV) containing 10% fetal bovine serum (FBS; Tissue Culture Biologicals; catalog no. 101) at 37°C and 5% CO_2_.

### Plasmids.

For bacterial expression and purification, a gBlocks gene fragment (Integrated DNA Technologies, Inc.) coding for 6×His/P_ΔOD_ was cloned in a pET16b vector (16). To rescue recombinant VSV, pVSV1^+^-eGFP-P_ΔOD_ was derived from pVSV1^+^-eGFP (39). pVSV1^+^-eGFP and a gBlocks gene fragment containing the sequence of the end of the N gene, the N-P intergenic region, and the P_ΔOD_ gene were digested with AloI and BstZI17I restriction enzymes and ligated together.

pVSV1^+^-eGFP/P_Δ_OD was derived from pVSV1^+^-eGFP/P (40) after amplification of the P_ΔOD_ gene from pVSV1^+^-eGFP-P_ΔOD_ and insertion by ligation.

### Viruses.

VSV-eGFP-P_WT_ and VSV-eGFP/P_WT_ were described previously (39, 40). VSV-eGFP-P_ΔOD_ and VSV-eGFP/P_ΔOD_ were rescued following previously described protocol (41) using pVSV1^+^-eGFP-P_ΔOD_ and pVSV1^+^-eGFP/P_ΔOD_ plasmids, respectively. Virus stocks were grown on BSR-T7 cells, and we determined the titer by plaque assay on BSR-T7 or Vero cells. Briefly, cells were seeded in DMEM-10% FBS and infected 1 day later for 1 h with viruses at a multiplicity of infection (MOI) of 0.01. Virus suspensions were replaced by DMEM-2% FBS, and cell supernatants were harvested when 95% of the cells were infected and ready to detach (about 24 h for VSV-eGFP-P_WT_). For gradient-purified viruses, infected cell supernatant was first concentrated through a 15% sucrose cushion in NTE (10 mM Tris-HCl pH 7.4, 100 mM NaCl, and 1 mM EDTA) at 110,000 × *g* for 2 h at 4°C. Pellets were resuspended overnight at 4°C in NTE, put on top of a linear 15 to 45% sucrose gradient in NTE, and centrifuged at 200,000 × *g* for 3 h at 4°C. Bands corresponding to virus were collected in 0.5-ml tubes by side puncture of the tube and diluted 10 times in NTE.

### Proteins and nucleocapsid purification.

P and P_ΔOD_ were purified from BL21(DE3) Escherichia coli cells and the L protein from Spodoptera frugiperda 21 (Sf21) cells as previously described (16). Briefly, after cell lysis, proteins were affinity purified with HisTrap HP (Ge Healthcare) followed by gel filtration (Superdex 200 HR 10/30; GE Healthcare). P proteins were stored in 20 mM Tris (pH 7.4), 150 mM NaCl, and 1 mM dithiothreitol (DTT) buffer, and L proteins were stored in 50 mM Tris-HCl (pH 7.4), 200 mM NaCl, 15% glycerol, and 1 mM DTT buffer. N-RNA templates were purified from gradient-purified VSV-eGFP-P_ΔOD_ virions as previously described (42).

### Size exclusion chromatography with multiangle light scattering.

SEC-MALS analysis was performed on an Agilent 1260 Infinity liquid chromatography system in phosphate-buffered saline (PBS) by use of a Wyatt Dawn Heleos II multiangle light scattering detector and Optilab T-rEX refractive index detector at the Center for Macromolecular Interactions, Harvard Medical School. Data were processed using Astra 7, and weight-averaged molar mass was fit using the Zimm method and a protein refractive index increment of 0.185. Fitting errors of 0.7% and 1.4% were achieved for P_WT_ and P_ΔOD_, respectively.

### *In vitro* RNA synthesis assays.

*In vitro* RNA synthesis assays on naked RNA were performed as previously described (21) using purified L protein and either P_WT_ or P_ΔOD_. Transcription assays on encapsidated RNA were performed using N-RNA extracted from VSV-eGFP-P_ΔOD_ virions and purified P_WT_, P_ΔOD_, and L proteins (42).

### Analysis of primary transcripts.

BSR-T7 cells were seeded in 6-well plates and incubated 1 day later in phosphate-free DMEM (Gibco; catalog no. 11971-025) for 30 min followed by a 30-min incubation in phosphate-free DMEM containing 10 μg/ml actinomycin D (Sigma; catalog no. A5156) and 100 μg/ml cycloheximide (VWR; catalog no. 94271). Cells were then infected for 30 min with sucrose cushion-purified virus at an MOI of 100. Virus solutions were replaced by 1 ml phosphate-free DMEM containing 10 μg/ml actinomycin D, 100 μg/ml cycloheximide, and 10 μl of phosphorus-32 radionuclide (PerkinElmer; catalog no. NEX053H005MC). At 2, 3, 4, 5, and 6 h postinfection, RNA was extracted using TRIzol reagent (Invitrogen; catalog no. 15596018) following the manufacturer’s protocol. RNA was boiled at 100°C for 1 min, incubated on ice for 2 min, mixed with a 1.33× loading buffer (33.3 mM citrate pH 3, 8 M urea, 20% sucrose, and 0.001% bromophenol blue), and analyzed on a 25 mM citrate, pH 3, 1.75% agarose, 6 M urea gel run for 18 h at 4°C and 180 V. Gels were fixed (in 30% methanol and 10% acetic acid), dried, and exposed overnight to a phosphor screen (GE Healthcare), and the radiolabeled RNA products were visualized using a Typhoon FLA 9500 scanner (GE Healthcare).

### Analysis of DI-T replication.

BSR-T7 cells were seeded in 6-well plates and infected 1 day later with a recombinant vaccinia virus expressing the T7 polymerase (vTF7-3) and DI-T particles for 1 h at 37°C in Dulbecco’s PBS (DPBS; Sigma; catalog no. 59300C). Cells were then transfected using Lipofectamine 2000 with plasmids expressing L (0.24 μg), N (2.9 μg or indicated amounts), and P or P_ΔOD_ (0.8 μg). Five hours later or at indicated hours, the medium was removed, and cells were incubated in 1 ml phosphate-free DMEM containing 10 μg/ml actinomycin D for 30 min before the addition of 10 μl of phosphorus-32 radionuclide. Cells were incubated for 3 h at 37°C before RNA was harvested by the use of TRIzol reagent following the manufacturer’s protocol. RNA was boiled at 100°C for 1 min, incubated on ice for 2 min, mixed with a 1.33× loading buffer (33.3 mM citrate pH 3, 8 M urea, 20% sucrose, and 0.001% bromophenol blue), and analyzed on a 25 mM citrate, pH 3, 1.75% agarose, and 6 M urea gel run for 18 h at 4°C and 180 V. Gels were fixed (in 30% methanol and 10% acetic acid), dried, and exposed overnight to a phosphor screen (GE Healthcare), and the radiolabeled RNA products were visualized using a Typhoon FLA 9500 scanner (GE Healthcare). DI-T band intensities were quantified with ImageJ software.

### FRAP of viral replication compartments.

Vero cells were plated at 3 × 10^5^ cells per chamber in a 8-well chambered cover glass (Cellvis) and infected 20 and 24 h later at an MOI of 3 by VSV-eGFP/P_ΔOD_ and VSV-eGFP/P_WT_, respectively. Thirty hours after seeding, the photobleaching was performed using the Vector photomanipulation module attached to a Marianas spinning disk confocal platform (3i, Denver, Colorado). The images were acquired using a Plan-Apochromat 100×/1.4 oil lens (Carl Zeiss, Jena, Germany). The incubation system (Okolab, Naples, Italy) was set at 5% CO_2_ and 37°C. Four images were acquired prior to bleaching and imaging with the 488-nm laser.

For data analysis, mean background fluorescence was measured from an area outside the cells and subtracted from other measurements. Mean fluorescence intensities of each photobleached area were also corrected for the photobleaching that occurred during image acquisition postbleach and normalized by the average fluorescence prebleach. Photobleaching postbleach was measured on nonbleached compartments. After normalization, mean recovery was calculated using EasyFRAP (43) and fit with a double-exponential model: *Y*(*t*) = *Y*_0_ + *A*_fast_(1 − *e*^−^*^K^*^fast^*^t^) + *A*_slow_(1 − *e*^−^*^K^*^slow^*^t^).
